# Iontophoretic Drug Delivery in the Oral Cavity

**DOI:** 10.3390/pharmaceutics10030121

**Published:** 2018-08-07

**Authors:** Apipa Wanasathop, S. Kevin Li

**Affiliations:** Division of Pharmaceutical Sciences, James L Winkle College of Pharmacy, University of Cincinnati, 231 Albert Sabin Way, MSB # 3005, Cincinnati, OH 45267-0514, USA; wanasaaa@mail.uc.edu

**Keywords:** iontophoresis, drug delivery, transport, enamel, dentin, buccal, gingiva, palate, oral cavity

## Abstract

Iontophoresis is a noninvasive method to enhance systemic and local drug delivery by the application of an electric field. For systemic drug delivery in the oral cavity, iontophoresis was studied primarily for transbuccal delivery. Significant enhancement of drug delivery was observed in buccal iontophoresis compared to passive transport for different drugs. For local drug delivery in the oral cavity, iontophoresis could enhance drug penetration into the enamel, dentin, and other oral tissues for the treatment of oral diseases. Iontophoresis was evaluated in dentistry such as to produce local anesthesia and treat tooth decalcification and hypersensitivity, but this technology has not been fully utilized. The most common drugs in these evaluations were fluoride and lidocaine. In general, there is limited knowledge of the mechanisms of iontophoresis in the oral tissues. In vivo animal and human studies have suggested that iontophoresis is safe in the oral cavity under the conditions investigated. The present review covers the topics of iontophoretic drug delivery in the oral cavity for both systemic and local treatments. The anatomy and diseases in the oral cavity for iontophoretic drug delivery are also briefly reviewed, and the challenges for this drug delivery method are discussed.

## 1. Introduction

Drug delivery in the oral cavity can be divided into two main categories: systemic and local drug delivery. Effective drug delivery in the oral cavity is essential for the treatment of oral diseases in both soft and hard oral tissues (e.g., mucosal and enamel, respectively). Systemic drug delivery via the oral mucosal route such as buccal delivery is an alternative route to gastrointestinal (GI) absorption. However, there are limited studies on the mechanisms of drug delivery in oral disease treatments. For example, there is little knowledge of drug permeation for the gingiva with no mechanistic studies of gingival drug delivery. As the advancement in drug delivery technologies relies heavily on our understanding of these topics, effective local drug delivery to the tissues in the oral cavity lags behind other routes of administration. Particularly, drug delivery and technologies such as iontophoresis have been developed and well characterized for other routes of administration such as skin. Drug delivery knowledge in the oral cavity, particularly for the treatments of oral diseases, has been lacking. The present review covers the topics of iontophoretic drug delivery in the oral cavity for both systemic and local treatments. The perspective and challenges on iontophoretic drug delivery in these areas are also discussed. 

## 2. Anatomy

The oral cavity is a part of the upper portion of the aerodigestive tract. It consists of the lips, oral mucosa, teeth, the hard palate, and the anterior two thirds of the oral tongue [1]. Each area has different structures and characteristics, to provide different functions. This leads to the differences in barrier properties and permeabilities. The structures and physiologies of the teeth, buccal, sublingual, and gingiva will be reviewed to gain better understanding in drug delivery to the oral cavity.

**Figure 1:**
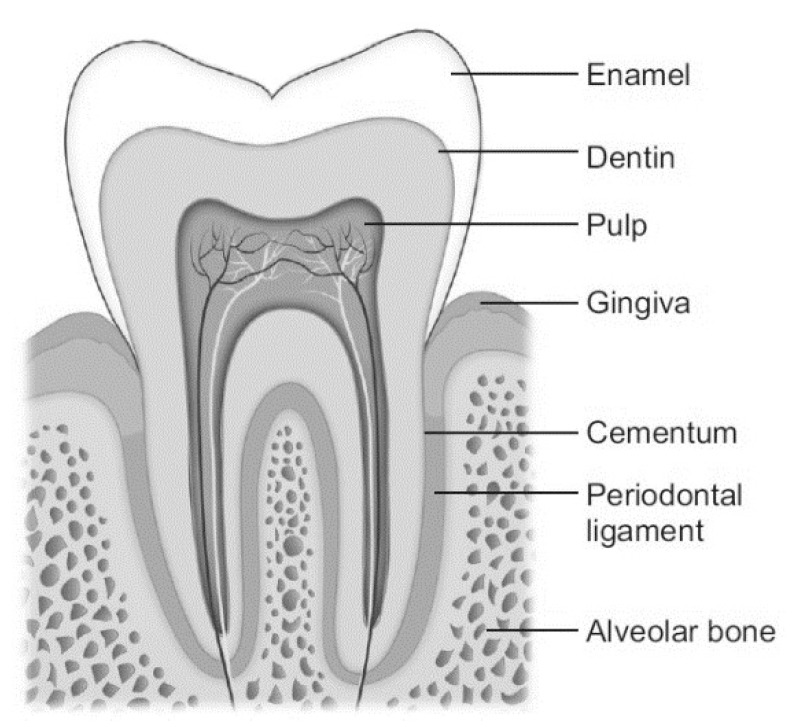
Structure of a tooth and periodontium. Modified from reference [4], with permission from Radiologic Clinics of North America, published by Elsevier, 2018.

Teeth are composed of dental tissues that can be divided into two main groups: mineralized hard and non-mineralized soft tissues. The mineralized hard tissues are enamel, dentin, and cementum. The non-mineralized soft tissue is the pulp. Figure 1 shows the structure of a tooth and its periodontium. Enamel is the most mineralized substance in the human body and covers the outer aspect of the crown of a tooth. Enamel is composed mainly of hydroxyapatite, a crystalline calcium phosphate with a low solubility product constant [2]. Dentin is the layer underneath the enamel. It is less calcified than the enamel and makes up the bulk of the crown and roots. It also surrounds the pulpal tissues within the tooth. When the dentin is exposed, the dentinal fluid flows outward through dentinal tubules from the pulp [3]. Cementum is the tissue layer that covers the roots of a tooth and surrounds the underlying dentin. The cementoenamel junction is where the enamel and cementum meet. It marks the boundary between the crown and the roots of the tooth. The pulp is a soft tissue composed of nerves and vessels and fills the internal cavity of a tooth. It delivers the nutrition, provides sensory function, and controls the blood flow [4]. The supporting structure of the teeth is called periodontium. The periodontium includes the periodontal ligament, gingival tissue, bone, blood, and nerves around the teeth. The periodontal ligaments are groups of fibers that act as a shock-absorber for the teeth and hold the cementum to the bone [5]. The thickness of each part of the tooth differs from species to species and from person to person. Human premolars have average enamel thickness of 1.39 mm and dentin thickness of 1.79 mm [6]. 

The oral mucosa consists of the buccal, sublingual, and gingival mucosa in the oral cavity. It is usually covered with saliva, creating a moist surface. The oral mucosa is a complex series of tissues that lined with layers of stratified squamous epithelium and basement membrane, supported by a connective tissue lamina propria. Systemic drug delivery can be achieved through a network of arteries and capillaries in the oral mucosa [7]. 

The buccal mucosa is the area inside the cheek and between the gums and lower lips. It has an average surface area of ~100 cm^2^ in the oral cavity of human adults and is approximately 0.5–0.8 mm thick [7]. The tissue complex consists of the outer epithelium and basement membrane, supported by the underneath connective tissue. The basement membrane is approximately 1–2 µm thick and is a continuous layer of extracellular material. Layers of non-keratinized stratified squamous epithelium form the outer buccal epithelium, which contains mostly phospholipids [8]. It also contains low molecular weight proteins in the form of tonofilament. The cells in these outer layers have their organelles and cytoplasmic function, which is different from keratinized cells that will be described later. The basal layer of the epithelium is mitotically active to differentiate into replacement cells that are shed from the outermost buccal surface [7]. A function of the buccal mucosa is to protect the underlying tissue from physical damages and entry of foreign substances in the oral cavity. The non-keratinized cells also provide the elasticity and flexibility, which allows the tissue to accommodate chewing, swallowing, and speaking [9].

The sublingual mucosa is similar to the buccal mucosa but is thinner (around 0.1–0.2 mm thick) with less layers of epithelial cells. The epithelial cells are stratified and non-keratinized [8]. The thinner epithelium is expected to be more permeable than the buccal mucosa [10]. 

**Figure 2:**
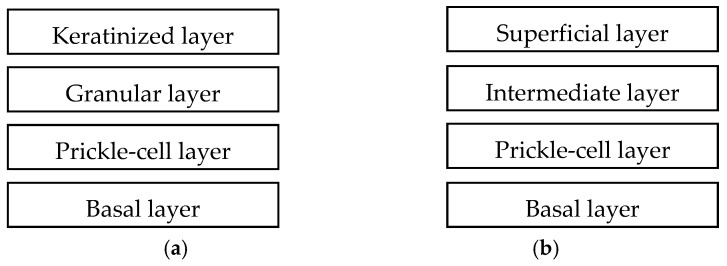
Structures of (**a**) keratinized and (**b**) non-keratinized oral mucosa [10].

The gingival mucosa is the mucosal lining of the tissue that covers the necks of the teeth and the alveolar bones. It joins the buccal mucosa at the gingivobuccal sulcus [1]. The gingiva and hard palate are lined with keratinized cornified epithelium surface called masticatory mucosa. The keratinized cells are flattened and do not have any organelles [7]. Figure 2 illustrates the structures of keratinized and non-keratinized oral mucosae. The gingival mucosa is approximately 0.2–0.25 mm thick [11,12]. The thickness of whole gingiva is approximately 1–1.5 mm [13,14]. The keratinized cells are tightly attached to the underlying collagenous connective tissue, allowing the epithelium surface to handle the mechanical forces related with the mastication [9]. These keratinized cells contain high molecular weight keratin and neutral lipids such as ceramides and acyl ceramides [15]. The permeability of the gingiva membranes is less than the buccal and sublingual mucosa [10]. 

## 3. Oral Diseases

Common oral diseases include dental caries, tooth hypersensitivity, and periodontal diseases. Dental caries are damages to a tooth caused by acids from decay-causing bacteria in the mouth. The acids dissolve the enamel on the surface creating holes (cavities) in the tooth. 

Tooth decalcification is due to the loss of calcium in the enamel. Enamel decalcification appears as white spots on the teeth. These weak spots are the early sign of tooth decay. Young children have higher risk than adults due to early childhood caries. Tooth decay is largely preventable, but if left untreated can cause pain, infection, and even tooth loss. Tooth decay can be prevented by tooth brushing, flossing, and disruption of the bacteria on the tooth surface, sealant application, and topical fluoride. Fluoride diffuses into enamel to form fluoridated apatite, which has a lower solubility in an acid environment than hydroxyapatite mineral, and strengthen the enamel before cavities are developed. The presence of fluoride also provides stronger enamel in the tooth remineralization process and contributes towards restoring the strength of the tooth structure. Enamel with surface erosion, e.g., by acid to form cavities, cannot be recalcified [16]. Tooth cavities are commonly treated by fillings. When the decay is severe, a crown that covers the top of the tooth rather than a filling is used to repair the damage. 

Tooth hypersensitivity occurs when the enamel that protects the teeth becomes thin or when gum recession exposes the underlying dentin. When the dentin is exposed, it responds to stimuli such as thermal, osmotic, chemical, and electrical stimulants. The dentin contains micron-size tubular structures that connect to the pulp. Changes in the flow of biological fluid in the dentinal tubules due to the stimulants can trigger sensation to the nerves in the pulp. Repeated stimuli to the hypersensitive dentin can also cause irritation to the pulp and inflammation. Although hypersensitivity can occur when the root of the tooth or dentin is exposed, not all exposed dentin surfaces cause dentin hypersensitivity. Tooth hypersensitivity can be treated by the occlusion of dentin tubules using resins, varnishes, and toothpastes (e.g., Sn containing toothpastes) or desensitization of the nerve using potassium ion. These treatments can be applied in dental offices or in-home uses with over-the-counter or prescription products. 

Periodontal diseases are related to infections to the gum and bone tissues around the teeth. The diseases can lead to tooth loss and complications such as osteomyelitis and facial cellulitis. Gingivitis is the inflammation or infection of the gums in the early stages of periodontal diseases. Gingivitis can lead to periodontitis, the inflammation and infection spreading from the gingiva to the ligaments and bones around the teeth. Bacterial toxins and immune response to the infection break down the bone and connective tissues that support the teeth. Chronic periodontitis shows a strong association with cardiovascular disease and type 2 diabetes mellitus. The potential of bacteria invasion from the periodontal areas to systemic health is also a concern in advanced stages of periodontitis. Current treatments of periodontal diseases are scaling, root planing, and surgical procedure such as flap surgery and bone grafts. Sustained drug delivery systems of antibacterial and anti-inflammatory have also been used in the treatments of periodontal diseases. 

## 4. Systemic Drug Delivery in the Oral Cavity

Buccal drug delivery is the most common form of systemic drug delivery in the oral cavity. Examples of buccal drug delivery systems are tablet, spray, mucoadhesive, sublingual lozenge, chewing gum, film, and oromucosal solution. Compared to systemic administration via the GI tract, the advantages of buccal delivery include the bypass of hepatic first-pass effect, no interference from acidity and enzyme relative to the GI tract, ease of dosing due to the accessibility of the oral cavity, and easy removal of the drug delivery system in the event of adverse reactions. Once the drug penetrates the epithelium of the buccal membrane, it enters the systemic circulation via the vascularized tissue and the jugular vein. Although the permeability of the buccal mucosal membrane is higher than that of cornified tissues such as the stratum corneum [17], it is low compared to that in GI absorption (the GI mucosal monolayer). Saliva secretion can dilute drug absorption when the dosage form is not completely sealed from the environment. Mathematical transport models have been examined for buccal absorption [18]. Experimental methods to evaluate buccal drug delivery in vitro and in vivo have been recently reviewed [19] and standardized methodologies that are biorelevant for the evaluation and prediction of the performance of buccal drug delivery systems are needed. Technologies such as chemical enhancers, polymeric hydrogel, and iontophoresis were utilized to enhance the delivery of macromolecules such as peptides and proteins across buccal mucosa [20,21]. Chemical penetration enhancers including surfactants, bile salts, and fatty acids have been previously reviewed for buccal drug delivery [22]. Physical penetration enhancers have also been used, and the focus of the present review is iontophoresis. 

## 5. Local Drug Delivery in the Oral Cavity

Treatment of local diseases by systemic drug administration generally requires a high dose of a drug to achieve its therapeutic concentration at the target tissues, and this can result in high systemic drug concentration. Local treatment is more effective and can prevent systemic adverse effects. For example, mouth-rinses are a common form of topical administration for local drug delivery to the oral cavity in the prevention of periodontal diseases [23,24,25]. Topical anesthesia is commonly used to reduce pain in the treatment of oral lesion, for discomfort, and in dentistry [26]. However, existing local administration in the oral cavity is not very effective because (a) the drug is easily diluted and rapidly eliminated by the saliva; (b) drug distribution is not easily controlled to achieve therapeutic concentration at the target site; and (c) drug permeation across the membrane barrier can be poor. To overcome these shortcomings, technologies such as bioadhesive polymers, gels, implants, and microspheres have been used to prolong the release and residence time of the drug at their sites of administration (e.g., PerioChip, Atridox, Actisite, Elyzol, and Arestin for local drug delivery to treat periodontal diseases) [27,28,29]. Iontophoretic drug delivery has also been used in dentistry and oral care. For example, iontophoresis of fluoride and peroxide for the enamel can be used to treat decalcification (e.g., in smooth surface decay) and teeth whitening, respectively. Local anesthetics can be applied to the oral mucosal tissues using iontophoresis before oral procedure. Iontophoresis of antibacterial and anti-inflammatory agents to the gingiva and surrounding tissues can be used to prevent systemic bacteria invasion and control periodontal disease progression. 

## 6. Iontophoretic Drug Delivery

Iontophoresis enhances the delivery of both charged and uncharged drugs by the application of an electric current across a membrane. The mechanisms of iontophoresis include electrophoresis (direct-field effect), electroosmosis (electroosmotic solvent flow), and electropermeabilization (electroporation) [30,31,32,33,34]. Electrophoresis is based on the principle that positively charged ions are repelled from the anode and attracted to the cathode and negatively charged ions from the cathode to anode. Electrophoresis plays a major role in the enhanced transport of ionic drugs. Electroosmosis can enhance the transport of neutral and ionic drugs. In electroosmosis, both neutral and charged molecules are transported along the bulk solvent flow created by the electric field across a charged membrane. Electropermeabilization alters the barrier of a membrane under the influence of an electric field. It increases the intrinsic permeability of the membrane (e.g., membrane porosity) and alters the properties of the permeation pathways across the membrane (e.g., membrane pore charges and sizes). 

**Figure 3:**
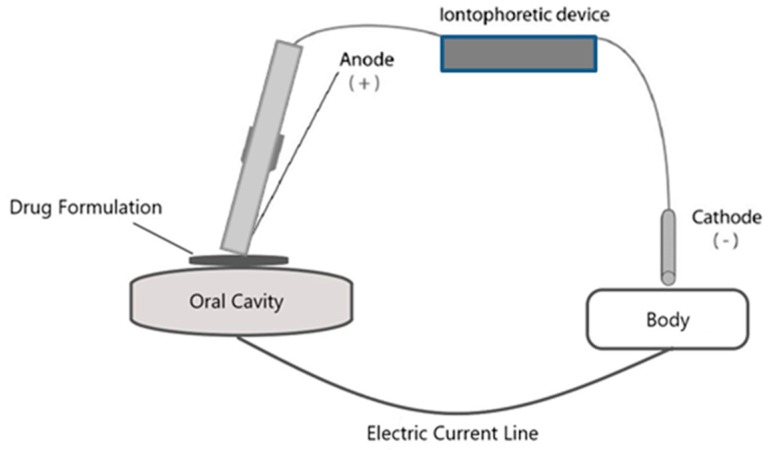
Schematic drawing of an iontophoretic device for oral cavity. Modified from reference [58].

Iontophoretic drug delivery is generally considered safe and noninvasive such as in transdermal and ocular iontophoresis. For example, the US Food and Drug Administration (FDA) has approved iontophoresis treatments such as transdermal fentanyl (Ionsys in 2015, Medicines Co., Parsippany, NJ, USA) [35,36] and sumatriptan (Zecuity in 2013, NuPathe/Teva) [37,38], topical pilocarpine for sweat stimulation (e.g., Nanoduct and Macroduct) [39,40], topical lidocaine for local skin anesthesia (e.g., Iontocaine and Lidosite) [41,42], and transtympanic lidocaine for tympanic membrane anesthesia [43,44]. Reverse transdermal iontophoresis for glucose monitoring was also approved [45]. There are clinical trials in ocular iontophoresis [46,47] and iontophoresis of off-label use with dexamethasone (using dexamethasone phosphate) for rehabilitation in physical therapy [48]. Iontophoresis is also used in hyperhidrosis treatment [49]. In iontophoresis, the drug-delivering electrode is placed in electrical contact with the site of administration and the return electrode is placed on an adjacent site or another body location to complete the electric circuit, as shown in the schematic drawing in Figure 3. A device is then connected to these electrodes that typically delivers up to 4 mA (or 0.5 mA/cm^2^) for skin delivery. For the ear and the eye, electric current up to 1 mA (~1.6 mA/cm^2^) and 3–3.5 mA (~3.7 mA/cm^2^) has been tested for transtympanic [44] and ocular [50,51] iontophoresis, respectively. In hyperhidrosis treatment, an electric current is applied and flows from one hand/foot to another, e.g., at 15–20 mA for 20–30 min, 3 times per week for 4 weeks [52,53,54]. The mechanisms of iontophoretic transport for transdermal (skin), ocular (cornea and sclera), and transungual (nail) have been systematically investigated in the literature [31,34,55,56,57]. However, iontophoresis for the oral cavity is not studied as well as those of the skin, eye, and nail. 

## 7. Iontophoresis on Oral Mucosa

### 7.1. Buccal Iontophoresis

Transmucosal iontophoresis via the buccal route provides an alternative method of systemic drug administration. The barrier properties, environment, and blood supply of the buccal area make buccal iontophoretic delivery an attractive approach for drugs that undergo first-pass metabolism or drugs that cannot be absorbed through the GI tract. Direct-field effect and electroosmosis were proposed as the main mechanisms in buccal iontophoretic transport [59]. Iontophoresis for buccal drug delivery was suggested to be safe under the conditions investigated in the animal and human studies in vivo [60,61]. In these studies, no adverse effects were observed after iontophoresis treatments using a transbuccal delivery device (IntelliDrug) that delivered 0.21 mA (2 mA/cm^2^) for 10 min, and histological analysis revealed few marked alterations in the buccal mucosa. 

Examples of drugs investigated for buccal iontophoresis are prilocaine and lidocaine for local anesthetics in dentistry [62], naltrexone for opiate addiction [60], and sumatriptan for migraine [63]. Other examples are the iontophoresis of atenolol [64], galantamine and naltrexone [65], 5-fluorouracil and leucovorin [66], and macromolecules such as dextrans [67]. These studies demonstrated enhanced drug delivery via iontophoresis (Table 1), suggesting the feasibility of this technique. In addition to drug delivery, reverse iontophoresis to extract analytes from the body has also been proposed for the buccal route in the monitoring of metabolites and endogenous substances [68]. Table 2 summarizes the buccal iontophoresis studies reviewed in this paper. 

**Figure 4:**
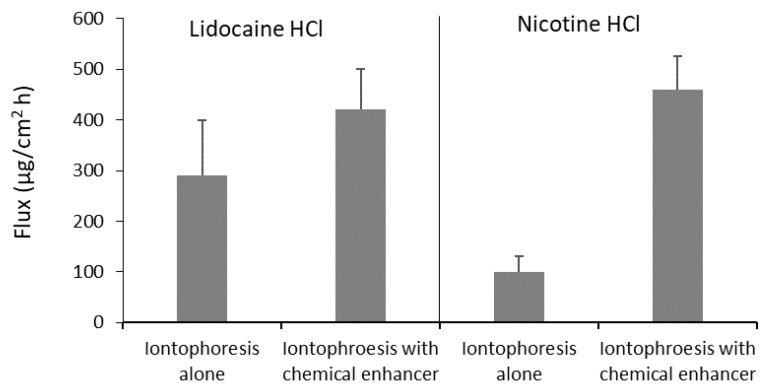
Lidocaine and nicotine fluxes of porcine buccal iontophoresis at 0.3 mA alone and with 5% dodecyl-2-(*N*,*N*-dimethylamino) propionate (DDAIP) as chemical enhancer. The combination of iontophoresis and chemical enhancer increases the fluxes of both drugs. Data from reference [73].

The synergetic effects of iontophoresis and chemical penetration enhancers have been evaluated and were found to be limited compared to the treatment of iontophoresis or enhancers alone for diltiazem, lidocaine, and nicotine [70] and ondansetron [76]. In another study, on the combined use of iontophoresis and chemical enhancers, Wei et al. investigated the effects of chemical enhancer pre-treatment on buccal iontophoretic delivery of lidocaine and nicotine and found that the chemical enhancers significantly enhanced iontophoretic delivery [73]. As an example, the data are shown in Figure 4. Oh et al. showed the combined effects of iontophoresis and enhancer sodium deoxyglycocholate but not *N*-acetyl-l-cysteine for buccal salmon calcitonin delivery in vitro [77]. In addition, they demonstrated the combination effects of iontophoresis and chemical enhancers on buccal delivery of salmon calcitonin in rabbits in vivo [78]. The animal study also showed a decrease in buccal thickness and minor tissue damage that recovered within 24 h after the removal of the iontophoresis electrodes.

### 7.2. Palate Iontophoresis

The organized intercellular lipids within the epithelium of buccal mucosa provide the major physical barrier in buccal drug delivery [17,88]. However, there have been limited studies on cornified oral mucosa such as the palate and gingiva, which have different anatomical structures compared to the buccal mucosa. In general, the cornified epithelium is less permeable compared to the buccal mucosa. To study the permeation mechanism of cornified oral mucosa, Ren et al. examined the barrier properties of palate for iontophoretic transport of tetraethylammonium, salicylate, mannitol, dexamethasone, fluoride, and chlorhexidine [69]. The results showed that the cornified epithelium was the rate-limiting barrier for drug delivery. It was also suggested that the direct-field effect was the dominant flux-enhancing mechanism in iontophoretic transport of ionic compounds. Electroosmosis was also found to contribute to the iontophoretic transport of both neutral and ionic permeants. Iontophoresis enhanced drug delivery into and across the palate and reduced the transport lag time. 

## 8. Iontophoresis on Enamel and Dentin

With the assistance of an external electric field, iontophoresis can facilitate the permeation of ionic and nonionic drugs into enamel and dentin. It has been suggested that iontophoresis treatments can control dental caries and dentin hypersensitivity. The use of an electric current for drug delivery and other dental applications is not new. This concept was introduced several decades ago in research and clinical practice [84,89,90] and in patent literature [91,92]. Iontophoresis application in the oral cavity is considered to be safe in humans [93]. For example, the treatments were performed at slowly increasing electric current to 0.5 mA or until the patient first feels a slight tingling sensation in the tooth to deliver a current dose of 1 mA-min to each tooth. Iontophoresis of higher current dose such as 1.0 mA and 5 min has also been used. The safety of iontophoresis was further supported by a recent study using an iontophoretic toothbrush that delivered electric current up to 0.4 mA for 2 min and showing that the iontophoretic application did not cause any adverse effects in the oral cavity in vivo [71]. Table 3 summarizes the iontophoresis studies (on enamel and dentin) reviewed in the present paper. 

**Figure 5:**
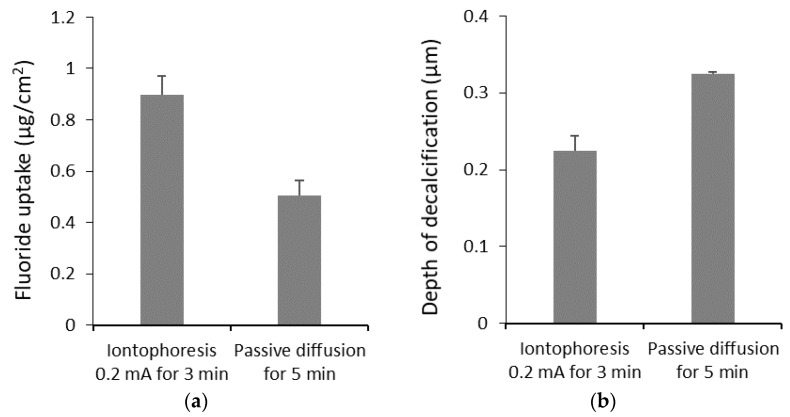
Comparing the fluoride (**a**) uptake and (**b**) depth of decalcification on bovine enamel between iontophoresis method and immersion method (passive diffusion). Iontophoresis could significantly increase the fluoride uptake and decrease the depth of decalcification. Data from reference [82].

Previous studies have suggested that iontophoresis can enhance the delivery of fluoride into enamel (Table 1). For example, in Figure 5, Tanaka et al. showed that iontophoresis could increase the uptake of fluoride and decrease decalcification by acid compared to those in the absence of iontophoresis [82]. However, Kim et al. found no significant difference in the reduction of lesion depth between iontophoresis-treated bovine incisors vs. conventionally applied fluoride, despite that iontophoresis increased the concentration of fluoride in the specimens [80]. Other studies have also suggested that the effect of fluoride iontophoresis on tooth remineralization was not superior to other fluoride application methods such as acidulated phosphate fluoride gel and sodium fluoride varnish [79,81]. Particularly, iontophoresis did not show any significant effects on the remineralization on initial carious lesions between treatment regimens compared to the other fluoride treatments, suggesting that remineralization likely occurred irrespective of the treatments despite that a higher amount of fluoride could be delivered into the enamel with iontophoresis. In addition to fluoride, iontophoresis was shown to enhance the penetration of iodide into enamel [87]. Ikeda et al. investigated the transport of lidocaine across human enamel with alternating current (AC) iontophoresis [72]. Chinnapareddy evaluated the relationship between the voltage and electric current for iontophoresis on enamel in humans in vivo [71]. To study the mechanisms of iontophoretic transport across enamel, Ren et al. examined the barrier and electrical properties of enamel [83]. It was shown that enamel is a resistive barrier for ions with permeability coefficients of ~10^−7^ cm/s (at thickness of ~0.5 mm) and transport pathways of neutral or low pore charge density and effective pore radii of approximately 0.7–0.9 nm. In the same study, iontophoresis of enamel for sodium fluoride, MFP, and SnF_2_, active ingredients in common dentifrice, was compared. 

For dentin, iontophoresis was examined using metronidazole, salicylate, and naproxen, and was found to provide enhanced delivery through intact and caries-affected dentin [75]. In addition to dental caries, fluoride iontophoresis was suggested to be a desensitizer for treatments related to cavity preparation, cementation of restorations, and enamel hypoplasia [96]. Iontophoresis was shown to enhance the delivery of fluoride into dentin [86]. Gupta et al. showed that iontophoresis of 2% NaF was more effective in reducing tooth sensitivity compared with topical fluoride applications [95]. Carlo et al. found that iontophoresis treatment of fluoride led to the desensitization in exposed dentinal lesions. The treatments reduced the sensitivity elicited by a blast of air and the touch of an explorer of ~90% patients [97]. Similar iontophoresis approaches have been examined for treating tooth hypersensitivity and desensitization [98]. It was suggested that iontophoresis of fluoride was a promising desensitizing method [99]. Aparna et al. compared the treatments of fluoride using acidulated phosphate gel iontophoresis and dentin-bonding agent application in reducing dentin hypersensitivity. Both methods were found to reduce hypersensitivity, and iontophoresis was more effective clinically and had fewer failures compared to the bonding agent application [94]. In addition to fluoride, iontophoretic delivery of lignocaine into exposed dentin was examined and found to be effective in anaesthetizing dentin-exposed teeth [58,74].

Besides penetration enhancement, iontophoresis treatment was suggested to be effective against biofilms in root canals [100]. Low voltage iontophoretic toothbrush to improve cleaning is commercially available (e.g., Ionic toothbrush system, HyG-2, Dyna-Dental Systems) [101]. The Ionic toothbrush was suggested to “shift” the charges of Staphylococcus aureus from negative to positive on the tooth surface, but the number of bacteria affected by 2-min tooth brushing was found to be small [102]. 

## 9. Conclusions: Challenges and Perspective

Studies of systemic and local drug delivery on soft and hard tissues in the oral cavity using iontophoresis have been reviewed. To our knowledge, these topics have not been reviewed together in the same paper. For both passive and iontophoretic drug delivery in general, the oral mucosa provides several advantages for systemic drug delivery over other routes of administration. For the treatment of oral diseases, local drug delivery to the tissues in the oral cavity has the advantage of direct drug application (e.g., topical application on the tissues) that can prevent systemic adverse effects. However, drugs are easily diluted and rapidly eliminated due to the flushing action of saliva, resulting in poor drug absorption in the tissues. There is also a lack of understanding of the barrier properties of these tissues in the oral cavity for drug delivery, and this is complicated by the different types of oral tissues (the soft and hard oral tissues) that have different anatomical structures. A quantitative structure permeation relationship (QSPR) to predict drug delivery in the oral tissues is not available for both passive and iontophoretic transport. The knowledge of barrier properties and mechanisms of membrane transport is essential for the advancement of drug delivery technologies such as iontophoresis. This has led to challenges in the development of effective drug delivery systems for the treatment of oral diseases. 

Iontophoresis enhances drug delivery by the application of an electric field across a membrane. This drug delivery method requires an electric current-generating element and electronics to create the electric field. The limited space in the oral cavity precludes the long-term use of a “bulky” iontophoretic device. This can also make the iontophoresis procedure inconvenient to patients and require the involvement of healthcare professionals to administer the iontophoresis treatments. The oral cavity is frequently used in normal activities such as eating and drinking, which creates a variable environment that disrupts drug delivery when long-duration drug application is needed. Therefore, iontophoresis application in the oral cavity is preferred to be short such as within minutes. This limitation can be challenging for effective drug delivery when the drugs have long transport lag times in the barriers of the oral tissues. 

Iontophoretic drug delivery is considered noninvasive and safe for short time uses. Iontophoresis has been used in dentistry and oral care, but this technology has not been fully utilized. Although studies have shown enhanced penetration of ions and drugs with iontophoresis for local delivery to the oral mucosa, enamel, and dentin, information on the mechanisms of iontophoretic transport for these tissues is limited. The development of an effective iontophoresis method (or an iontophoresis platform) for oral local drug delivery is significant as existing drug delivery methods for local treatment of oral diseases are not very convenient and effective. The topic of systemic iontophoretic drug delivery via the oral mucosa is also not well studied with only a handful of studies on buccal iontophoresis. In these studies, iontophoresis was shown to provide significant flux enhancement in buccal drug delivery. The advantages of systemic iontophoretic delivery from the oral cavity such as buccal iontophoresis vs. other systemic delivery methods and routes of administration have not been fully evaluated. 

Iontophoresis could be an effective drug delivery approach for the treatment of oral diseases such as tooth decalcification and hypersensitivity and periodontal diseases such as gingivitis and periodontitis. The limited knowledge of local drug delivery to these tissues and the lack of information on the mechanisms of iontophoretic drug delivery in the tissues is an obstacle in the advancement of iontophoresis for oral care. The development of iontophoretic delivery devices for the oral cavity is lagging behind other iontophoresis fields such as transdermal and ocular iontophoresis. Currently, there is no FDA approved iontophoretic drug delivery treatment for oral diseases in the dentist office and in-home treatment by patients. 

## Figures and Tables

**Table 1:** Effect and enhancement in drug delivery due to iontophoresis. ^a^

Permeant/Drug	Formulation	Tissue	Iontophoresis Condition	Iontophoretic Enhancement or Effect	Reference
3 kDa Dextran	Hydrogel (1 mg/mL)	Porcine buccal	0.1 mA 8 h	7.1	Patel et al. [67]
10 kDa Dextran	Hydrogel (1 mg/mL)	Porcine buccal	0.1 mA 8 h	32	
12 kDa Parvalbumin	Hydrogel (1 mg/mL)	Porcine buccal	0.1 mA 8 h	36	
5-Fluoruuracil	Solution (20 mM) Gel (10 mM)	Bovine buccal Bovine buccal	1 mA/cm^2^ 20 min 1 mA/cm^2^ 10 min	8 4	Gratieri and Kalia [66]
Atenolol	Solution (0.027–0.10 M)	Porcine buccal	0.1 mA/cm^2^ 8 h 0.2 mA/cm^2^ 8 h 0.3 mA/cm^2^ 8 h 0.4 mA/cm^2^ 8 h	6 18 36 58	Jacobsen [64]
Chlorhexidine	Solution (0.12%)	Bovine palate	0.5 mA 2 h	10	Ren et al. [69]
Diltiazem	Gel (2%)	Porcine buccal	0.3 mA 8 h	3 ^b^	Hu et al. [70]
Dexamethasone	Solution (10 µg/mL)	Bovine palate	0.1 mA 8 h	2.1	Ren et al. [69]
Eosyn Y dye	Solution (7.3 mM)	Human enamel	6 mA 60 min	Dye increase in the cracks	Chinnapareddy et al. [71]
Lidocaine	Solution (1, 2, 5, 10, 20%)	Human enamel/dentin	3 V, 1 kHz, 10 min	6 ^b^	Ikeda et al. [72]
Lidocaine	Solution (10 mg/mL)	Porcine buccal	0.5 mA/cm^2^ 6 h	4	Telò et al. [63]
Lidocaine	Gel	Porcine buccal	0.3 mA/cm^2^ 8 h with chemical enhancers	8 ^b^	Wei et al. [73]
Lidocaine	Gel (2.5%)	Porcine buccal	0.3 mA 8 h	5.4 ^b^	Hu et al. [70]
Lidocaine	Hydrogels (2.5% *w*/*w*)	Porcine buccal	1 mA/cm^2^ 1 h	4	Cubayachi et al. [62]
Leucovorin	Solution (20 mM) Gel (10 mM)	Bovine buccal Bovine buccal	1 mA/cm^2^ 20 min 1 mA/cm^2^ 10 min	3 3	Gratieri and Kalia [66]
Lignocaine with epinephrine	Solution (20% lidocaine, 0.1% epinephrine)	Human dentin	0.2 mA 2 min	Anesthetized 85% of participants	Smitayothin et al. [58]
Lignocaine with epinephrine	Solution (20% lidocaine, 0.1% epinephrine)	Human dentin	0.12 mA 90 s	Fully anesthetized for 40 min	Thongkukiatkun et al. [74]
Mannitol	Solution (1 mg/mL)	Bovine palate	0.1 mA 8 h	6.2	Ren et al. [69]
Metronidazole	Solution (0.05 M)	Human dentin	0.05 mA 10 min	2 ^b^	Puapichartdumrong et al. [75]
Nicotine	Gel	Porcine buccal	0.3 mA/cm^2^ 8 h	450 ^b^	Wei et al. [73]
Nicotine	Gel (2%)	Porcine buccal	0.3 mA 8 h	90 ^b^	Hu et al. [70]
Naproxen	Solution (0.05 M)	Human dentin	0.05 mA 10 min	2 ^b^	Puapichartdumrong et al. [75]
Ondansetron	Gel (0.5%)	Porcine buccal	0.1 mA 8 h 0.2 mA 8 h 0.3 mA 8 h	3.3 5.2 7.1	Hu et al. [76]
Prilocaine	Hydrogels (2.5%)	Porcine buccal	1 mA/cm^2^ 1 h	8.5	Cubayachi et al. [62]
Salmon calcitonin (sCT)	Solution (200 IU)	Porcine buccal	0.5 mA/cm^2^ 8 h	66	Oh et al. [77]
Salmon calcitonin (sCT)	Hydrogel (200 IU)	Rabbit buccal	0.5 mA/cm^2^	Hypocalcemic effect drop 20%	Oh et al. [78]
Sodium salicylate	Solution (0.15 M)	Bovine palate	0.1 mA 8 h	9.4	Ren et al. [69]
Sodium salicylate	Solution (0.05 M)	Human dentin	0.05 mA 10 min	2 ^b^	Puapichartdumrong et al. [75]
Sodium fluoride	Solution (0.05 M)	Bovine palate	0.1 mA 8 h	50 ^b^	Ren et al. [69]
Sodium fluoride	Solution (2%)	Bovine enamel	0.4 mA 4 min	No differences in remineralization	Kim et al. [79]
Sodium fluoride	Solution (2%)	Bovine enamel	0.3 mA 4 min for 5 days	16% increase in fluoride content, no difference in lesion depth	Kim et al. [80]
Sodium fluoride	Solution (2%)	Bovine enamel	0.2 mA 4 min	No differences in remineralization	Lee et al. [81]
Sodium fluoride	Solution (2%)	Bovine enamel	0.5 mA 3 min 0.5 mA 5 min 0.5 mA 10 min	1.6 1.9 1.5	Tanaka et al. [82]
Sodium fluoride	Solution (1000 ppm) as NaF, MFP, or SnF_2_	Bovine enamel	0.1 mA 8 h	60–80	Ren et al. [83]
Sodium fluoride	Solution (4%)	Rat enamel	0.5–0.6 mA 30 s	Double resistivity to acid	Wagner et al. [84]
Sodium fluoride	Gel (1.1%)	Human enamel	10 mA-min	Increase fluoride retention from 34 to 52%	Barbakow et al. [85]
Sodium fluoride	Gel (0.33%)	Human dentin	1.5% mA 3 min	Close 50% open tubules	Patil et al. [86]
Sodium fluoride	Acidulated phosphate fluoride gel (1.23%)	Bovine enamel	0.4 mA 4 min	No differences in remineralization	Kim et al. [79]
Sodium fluoride	Acidulated phosphate fluoride gel (1.23%)	Bovine enamel	0.2 mA 4 min	No differences in remineralization	Lee et al. [81]
Sodium iodide	Solution (0.04 M)	Human enamel	1–12.5 V 15 min–24 h	3.2	Stowell and Taylor [87]
Sumatriptan succinate	Solution (33 mM)	Porcine buccal	0.75 mA/cm^2^ 2 h	16	Telò et al. [63]
Tetraethylammonium	Solution (0.15 M)	Bovine palate	0.1 mA 8 h	42	Ren et al. [69]

^a^ This table is not intended to provide a complete list of all studies but main findings reviewed in this paper; ^b^ Estimated values from data presented in the cited reference figures.

**Table 2:** Summary of studies on iontophoresis in soft tissue in the oral cavity. ^a^

System	Membrane	Drug/Permeant	Enhancer	Reference
In vitro, porcine	Non-cornified, Buccal	Lidocaine, nicotine	0.3 mA/cm^2^ for 8 h; and Pre-treatment with dodecyl-2-(*N*,*N*-dimethylamino) propionate hydrochloride, *N*-(4-bromobenzoyl)-*S*,*S*-dimethyliminosulfurane, azone, and propylene glycol	Wei et al. [73]
In vitro, porcine	Non-cornified, Buccal	Lidocaine, prilocaine	1 mA/cm^2^ for 1 h	Cubayachi et al. [62]
In vitro, porcine	Non-cornified, Buccal	Diltiazem, lidocaine, nicotine	0.1 mA or 0.3 mA for 8 h; or Pre-treatment with dodecyl-2-(*N*,*N*-dimethylamino) propionate hydrochloride, *N*-(4-bromobenzoyl)-*S*,*S*-dimethyliminosulfurane, and azone	Hu et al. [70]
In vitro, porcine	Non-cornified, Buccal	Ondansetron	0.1, 0.2 and 0.3 mA for 8 h; or Pre-treatment with dodecyl 2-(*N*,*N*-dimethylamino) propionate, *N*-(4-bromobenzoyl)-*S*,*S*-dimethyliminosulfurane, azone	Hu et al. [76]
In vitro, porcine	Non-cornified, Buccal	Atenolol	0.1, 0.2, 0.3 and 0.4 mA/cm^2^ for 8 h	Jacobsen [64]
In vitro, porcine	Non-cornified, Buccal	Salmon calcitonin	0.5 mA/cm^2^; and *N*-acetyl-l-cysteine, deoxyglycocholate, or ethanol for 8 h	Oh et al. [77]
In vitro, porcine	Non-cornified, Buccal	Galantamine, naltrexone	0.4–1.5 mA/cm^2^	Mościcka-Studzińska et al. [65]
In vitro, porcine	Non-cornified, Buccal	Sodium dodecyl sulfate, urea as water promoter	0.25–1.0 mA/cm^2^	Mościcka-Studzińska et al. [59]
In vitro, porcine	Non-cornified, Buccal	Dextrans (3 and 10 kDa), parvalbumin (12 kDa)	0.1 mA for 8 h	Patel et al. [67]
In vitro, porcine	Non-cornified, Buccal	Dextrans (3 and 10 kDa), parvalbumin (12 kDa), bovine serum albumin (66 kDa)	0.1 mA for 8 h	Patel et al. [20]
In vitro, porcine	Non-cornified, esophageal epithelium	Sumatriptan succinate, lidocaine	0.38, 0.5, 0.75, 1.08, 1.5, 5.83 mA/cm^2^ for 2 h	Telò et al. [63]
In vitro, bovine	Non-cornified, Buccal	5-Fluorouracil, leucovorin	1 mA/cm^2^ for 10–20 min	Gratieri and Kalia [66]
In vitro, bovine	Cornified, Palate	Tetraethylammonium, salicylate, mannitol, dexamethasone, fluoride, chlorhexidine	0.1 mA for 8 h	Ren et al. [69]
In vivo, porcine	Non-cornified, Buccal	Naltrexone	2 mA/cm² for 10 min IntelliDrug device	Campisi et al. [60]
In vivo, rabbit	Non-cornified, Buccal	Salmon calcitonin and hypocalcemic effect was measured	0.5 mA/cm^2^; with *N*-acetyl-l-cysteine, deoxyglycocholate, or ethanol for 8 h	Oh et al. [78]

^a^ This table is not intended to provide a complete list of all studies but main findings reviewed in this paper.

**Table 3:** Summary of studies on iontophoresis in hard tissue in the oral cavity. ^a^

System	Membrane	Drug/Permeant	Enhancer	Reference
In vitro, bovine	Enamel	Fluoride for remineralization	0.4 mA, 12 V for 4 min	Kim et al. [79]
In vitro, bovine	Enamel	Fluoride for remineralization in early caries	0.1, 0.2, 0.3, 0.4 mA, 12 V for 4 min/day, 5 days	Kim et al. [80]
In vitro, bovine	Enamel	Fluoride for remineralization in early caries	0.2 mA for 4 min	Lee et al. [81]
In vitro, bovine	Enamel	Fluoride for dental caries prevention	0.2, 0.4, 0.5 mA for 3, 5, 10 min	Tanaka et al. [82]
In vitro, bovine	Enamel	Sodium fluoride, sodium monofluorophosphate, Tin (II) fluoride	0.1 mA for 8 h	Ren et al. [83]
In vitro, human	Enamel	Eosyn Y dye	0.7 and 6 mA for 60 min	Chinnapareddy [71]
In vitro, human	Enamel	Radiolabeled sodium iodide	1–12.5 V	Stowell and Taylor [87]
In vitro, human	Enamel	Lidocaine	AC-iontophoresis 3 V, 1 kHz	Ikeda and Suda [72]
In vitro, human	Dentin	Metronidazole, sodium salicylate, naproxen	0.05 mA for 10 min	Puapichartdumrong et al. [75]
In vitro, human	Dentin	Fluoride for tooth desensitization	1.5 mA for 3 min	Patil et al. [86]
In vivo, rat	Enamel	Fluoride for acid resistivity	0.5–0.6 mA for 30 s	Wagner et al. [84]
In vivo, human	Enamel	Fluoride for tooth desensitization	Increasing current until the patient felt a sensation	Aparna et al. [94]
In vivo, human	Enamel	Fluoride for dental caries prevention	3–5 mA for 2–3.3 min (10 mA min)	Barbakow et al. [85]
In vivo, human	Dentin	Lignocaine with epinephrine	0.2 mA for 2 min	Smitayothin et al. [58]
In vivo, human	Dentin	Lignocaine with epinephrine	0.12 mA for 90 s	Thongkukiatkun et al. [74]
In vivo, human	Dentin	Fluoride for post-operative desensitization	Variable current, current slightly below the level when the patient felt a sensation	Gupta et al. [95]

^a^ This table is not intended to provide a complete list of all studies but main findings reviewed in this paper.
